# Improving carrier mobility of polycrystalline Ge by Sn doping

**DOI:** 10.1038/s41598-018-33161-z

**Published:** 2018-10-04

**Authors:** Kenta Moto, Ryota Yoshimine, Takashi Suemasu, Kaoru Toko

**Affiliations:** 1 Institute of Applied Physics, University of Tsukuba, 1-1-1 Tennodai, Tsukuba, Ibaraki 305-8573 Japan; 2 JSPS Research Fellow, 8 Ichiban-cho, Chiyoda-ku, Tokyo 102-8472 Japan; 3 PRESTO, Japan Science and Technology Agency, 4-1-8 Honcho, Kawaguchi, Saitama 332-0012 Japan

## Abstract

To improve the performance of electronic devices, extensive research efforts have recently focused on the effect of incorporating Sn into Ge. In the present work, we investigate how Sn composition *x* (0 ≤ *x* ≤ 0.12) and deposition temperature *T*_d_ (50 ≤ *T*_d_ ≤ 200 °C) of the Ge_1−*x*_Sn_*x*_ precursor affect subsequent solid-phase crystallization. Upon incorporating 3.2% Sn, which is slightly above the solubility limit of Sn in Ge, the crystal grain size increases and the grain-boundary barrier decreases, which increases the hole mobility from 80 to 250 cm^2^/V s. Furthermore, at *T*_d_ = 125 °C, the hole mobility reaches 380 cm^2^/V s, which is tentatively attributed to the formation of a dense amorphous GeSn precursor. This is the highest hole mobility for semiconductor thin films on insulators formed below 500 °C. These results thus demonstrate the usefulness of Sn doping of polycrystalline Ge and the importance of temperature while incorporating Sn. These findings make it possible to fabricate advanced Ge-based devices including high-speed thin-film transistors.

## Introduction

Research on novel materials to replace Si has been actively pursued for sustainable improvement of electronic devices. Ge has attracted attention as the most promising candidate for next-generation material because it has a higher carrier mobility than Si for both electrons and holes and is compatible with conventional Si processing^1–3^. Effective mobilities in Ge metal-oxide-semiconductor field-effect transistors (MOSFETs) have exceeded those in Si-MOSFETs because of the development of device technologies including gate stacks^4–8^. In addition, Ge has a lower crystallization temperature and grain-boundary potential than Si^9–12^. These properties have motivated researchers to directly synthesize polycrystalline (poly-) Ge on various substrates, where the process temperature is usually limited, to fabricate advanced thin-film transistors (TFTs) for three-dimensional integrated circuits or high-performance mobile terminals^11,13^.

Many crystallization techniques have been developed, including solid-phase crystallization (SPC)^14–16^, laser annealing^17,18^, chemical vapor deposition^19,20^, flash-lamp annealing^21^, the seed layer technique^22^, and metal-induced crystallization^23–25^. By using these techniques, Ge-TFTs have been fabricated on thermally oxidized Si^21,26,27^, glass^28–31^, and even flexible substrates^22,32^. Since gate stack technology for Ge has developed sufficiently^8^, recent Ge-TFTs performance is limited by the properties of the poly-Ge thin film itself^21,22,26–32^. Some of these TFTs exhibited effective hole mobilities greater than 100 cm^2^/V s^22,26,28^. This value is equivalent to the effective mobility of bulk-Si p-MOSFETs; however, much lower than that of bulk-Ge p-MOSFETs^7,8^. Besides, leakage currents are still large due to the defects in the poly-Ge thin films. To further improve Ge-TFTs and put them into practical use, a simple way to form high-mobility, low-defect Ge thin films is strongly desired.

Recently, we reported that the deposition temperature *T*_d_ of the Ge precursor for SPC strongly influences the crystal quality and electrical properties of the resulting SPC-Ge^11^. This material has a hole mobility of over 300 cm^2^/V s, which is the highest mobility ever recorded for a thin film formed on insulators at temperatures below the melting point of Ge (937 °C). To further improve the electrical properties of the SPC-Ge, the present work incorporates small amount of Sn into Ge. GeSn itself has been studied as a material for high-speed transistors^33–37^ and photonic devices^37–40^. In addition, the effects of incorporating Sn into poly-Ge has been getting attentions^37^. The results indicate that the crystallization temperature is lowered^41–43^, the crystal grains are enlarged^44,45^, and the vacancy-induced accepters are reduced^46,47^. However, because of the poor properties of the original Ge, the electrical properties of the resulting GeSn are worse than our recent SPC-Ge^11^. The present study systematically investigates the effects of *T*_d_ and Sn concentration *x* in Ge_1−*x*_Sn_*x*_ (*x* ≤ 0.12) precursors for subsequent SPC and finds that the hole mobility dramatically increases from 80 to 380 cm^2^/V s.

## Results

**Figure 1:**
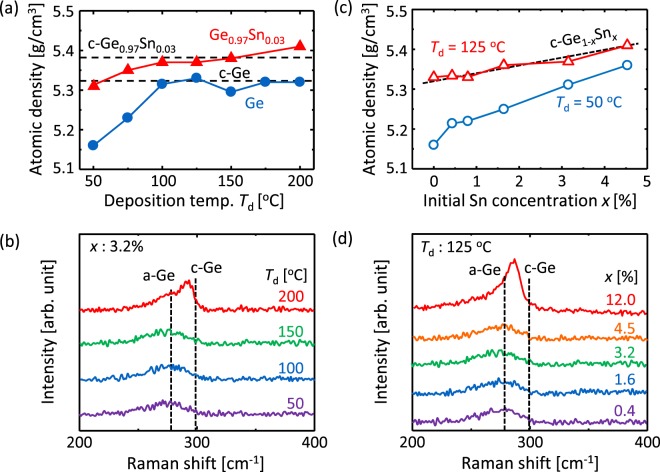
Characteristics of as-deposited Ge_1−*x*_Sn_*x*_ precursors. (**a**) Density of Ge and Ge_0.97_Sn_0.03_ (corresponding to *x* = 3.2%) as a function of *T*_d_, obtained from XRR patterns. The data for c-Ge and c-Ge_0.97_Sn_0.03_ are shown by the dotted lines. (**b**) Raman spectra for *x* = 3.2% with *T*_d_ = 50, 100, 150, and 200 °C. (**c**) Density of Ge_1−*x*_Sn_*x*_ with *T*_d_ = 50 and 125 °C as a function of *x*, obtained from XRR patterns. (**d**) Raman spectra for Ge_1−*x*_Sn_*x*_ samples for *T*_d_ = 125 °C with *x* = 0.4, 1.6, 3.2, 4.5, and 12.0%.

The as-deposited Ge_1−*x*_Sn_*x*_ layers, which are precursors for SPC, were analyzed by using x-ray reflectivity (XRR) and Raman spectroscopy. Figure 1(a) shows that, with increasing *T*_d_, the atomic density of both precursors Ge and Ge_0.97_Sn_0.03_ (corresponding to *x* = 3.2%) increases and asymptotically approaches that of crystals. In addition, for low *T*_d_ (≤100 °C), Sn doping allows the atomic density of the precursor approach that of the crystals. Figure 1(b) shows that Ge_0.97_Sn_0.03_ samples with *T*_d_ = 50, 100, and 150 °C exhibit broad peaks near 270 cm^−1^, corresponding to amorphous (a-) Ge. The sample with *T*_d_ = 200 °C exhibits a sharp peak near 300 cm^−1^, corresponding to crystalline (c-) Ge, in addition to an a-Ge peak. In this study, clear peaks corresponding to Sn-Sn or Ge-Sn vibrational modes^38^ were not observed because of the low *x* and/or the measurement condition of the Raman system. Figure 1(c) shows that the atomic density of the precursor with *T*_d_ = 50 and 125 °C increases with increasing initial Sn concentration *x*. Over the entire *x* range 0 ≤ *x* < 0.05, the atomic density for *T*_d_ = 125 °C exceeds that for *T*_d_ = 50 °C and is equivalent to that of crystalline GeSn. Figure 1(d) shows the *x* dependence of Raman spectra at *T*_d_ = 125 °C. The samples with *x* = 0.4%‒4.5% exhibit the a-Ge peak, whereas the sample with *x* = 12% exhibits both the crystalline Ge peak and the a-Ge peak. The study using transmission electron microscopy confirmed that the Ge layer with *T*_d_ = 125 °C is completely amorphous and contained no crystals. The crystallinity, defined as the ratio of the Raman peak intensity of c-Ge to that of a-Ge^11^, was found to be 56% for the Ge_0.97_Sn_0.03_ sample with *T*_d_ = 200 °C [Fig. 1(b)] and 69% for the Ge_0.88_Sn_0.12_ sample with *T*_d_ = 125 °C [Fig. 1(d)]. These results indicate that crystalline nuclei start to form in the a-Ge_1−*x*_Sn_*x*_ layer for *x* = 3.2% at *T*_d_ > 150 °C and *x* > 4.5% at *T*_d_ = 125 °C. This behavior is consistent with the previous reports that the crystallization of a-Ge_1−*x*_Sn_*x*_ is facilitated by increasing *x*^41‒43^ and *T*_d_^11^. Thus, these optical studies reveal that both *x* and *T*_d_ strongly influence the atomic density and crystalline state in the precursor layer.

**Figure 2:**
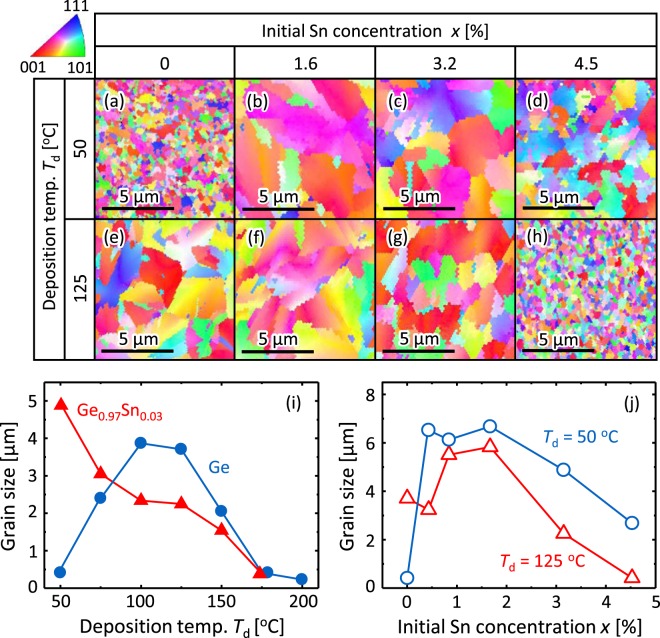
Grain size of SPC-Ge_1−*x*_Sn_*x*_ layers. (**a**–**h**) EBSD images organized as a matrix composed of *T*_d_ (50 and 125 °C) rows and *x* (0, 1.6, 3.2, and 4.5%) columns. Average grain size determined by EBSD for (**i**) Ge and Ge_0.97_Sn_0.03_ samples as a function of *T*_d_ and for (**j**) Ge_1−*x*_Sn_*x*_ samples for *T*_d_ = 50 and 125 °C as a function of *x*. Here, *T*_g_ = 450 °C.

The samples were then annealed for 5 h to induce SPC at a growth temperature *T*_g_ = 450 °C. Figures 2(a–j) show the crystal-orientation maps obtained by electron backscattering diffraction (EBSD), which indicate that the grain size dramatically varies with both *x* and *T*_d_. Figure 2(i) shows that, with respect to *T*_d_, the grain size evolves differently for Ge and GeSn. For Ge, the grain size increases with increasing *T*_d_ and then begins to decrease. As a result, the grain size peaks around 100 ≤ *T*_d_ ≤ 150 °C. Conversely, the grain size of Ge_0.97_Sn_0.03_ decreases with increasing *T*_d_. Note that the grain size of Ge_0.97_Sn_0.03_ greatly exceeds that of Ge when the substrate is not heated (*T*_d_ = 50 °C). Figure 2(j) shows that, for both *T*_d_ = 50 and 125 °C, the grain size of Ge_1−*x*_Sn_*x*_ strongly depends on *x* and peaks at *x* = 1.6%. For all samples containing Sn (*x* > 0), the grain size is larger at *T*_d_ = 50 °C than at *T*_d_ = 125 °C. The maximum grain size is approximately 7 µm, which is the largest grain size among semiconductor layers formed by SPC.

We investigate the origin of this evolution in grain size from the perspective of substitutional Sn concentration *y* in SPC-GeSn. Since the lattice constant of Ge_1−*y*_Sn_*y*_ depends on *y*, *y* can be determined from the Ge-Ge peak position in the Raman spectrum^34,38^. We therefore determine *y* from the Raman spectra by using the following equation proposed by Lin *et al*.^34^:

1 $${\rm{\Delta }}\omega (y)={\rm{ay}}+{\rm{\Delta }}{\omega }_{{\rm{strain}}}$$

where Δ*ω*(*y*) is the difference between the shift in the Ge-Ge peak of Ge_1−*y*_Sn_*y*_ [*ω*(*y*)] and that of the c-Ge wafer (*ω*_Ge_), *a* is a constant of 82 cm^−1^ ^34^, and Δ*ω*_strain_ is the shift due to strain. In general, the Ge-Ge peak of Ge [*ω*(0)] on a glass substrate shifts to the lower wavenumber than *ω*_Ge_ because of the strain induced by the difference between the thermal expansion coefficients of Ge and the glass substrate^14,44^. Assuming that the thermal strain of Ge_1−*y*_Sn_*y*_ is the same as that of Ge because *y* is low (<5%), Eq. (1) may be rewritten as

2 $$\Delta \omega (y)-\Delta {\omega }_{{\rm{strain}}}=({\omega }_{{\rm{Ge}}}-\omega (y))-({\omega }_{{\rm{Ge}}}-\omega (0))=\omega (0)-\omega (y)=ay$$

**Figure 3:**
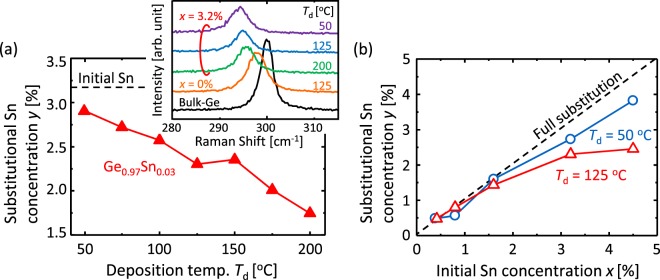
Substitutional Sn concentration *y* of SPC-Ge_1−*x*_Sn_*x*_ layers. (**a**) *y* for Ge_0.97_Sn_0.03_ samples as a function of *T*_d_ obtained from Raman spectra, some examples of which are shown in the inset. The dotted line corresponds to the initial Sn concentration (*x* = 3.2%). (**b**) *y* for Ge_1−*x*_Sn_*x*_ samples for *T*_d_ = 50 and 125 °C as a function of *x*. The dotted line shows the line when the initial Sn is fully substituted. Here, *T*_g_ = 450 °C.

Therefore, we estimate *y* from Raman spectra, of which examples are shown in the inset of Fig. 3(a). Figure 3(a) shows that *y* decreases with increasing *T*_d_. This suggests that higher *T*_d_ makes Sn precipitate, as estimated from the difference between *x* (=3.2%) and *y*. This result is likely caused by enhanced surface migration of Sn during precursor deposition. Figure 3(b) shows that *y* increases with increasing *x*, which is accompanied by Sn precipitation for *x* > 1.6%. This behavior can be explained from the perspective of the solid solubility of Sn in Ge (1–2%)^37^. It is well known that *y* exceeds the solid solubility for GeSn thin films grown in a non-equilibrium system including SPC^41–43^. The relationship between *y* and growth temperature in this study is approximately consistent with the previous reports. Considering that c-Sn facilitates Ge nucleation^42^, the decrease in grain size at higher *T*_d_ and for *x* > 1.6% [Fig. 2(i,j)] is attributed to the promotion of Ge nucleation because of Sn precipitation. In contrast, when Sn does not precipitate (*x* ≤ 1.6%), the grain size increases with increasing *x* [Fig. 2(j)]. The mechanism leading to this result remains unclear, but it may possibly be due to Sn doping weakening the amorphous bonds in Ge, which could enhance the lateral growth of crystals.

**Figure 4:**
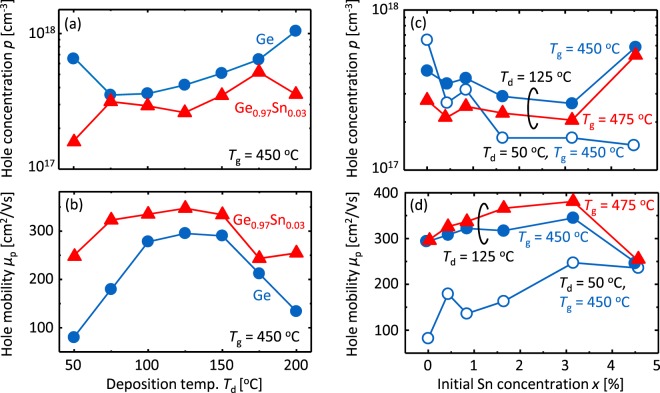
Electrical properties of the SPC-Ge_1−*x*_Sn_*x*_ layers. (**a**) Hole concentration *p* and (**b**) hole mobility *μ*_p_ for Ge and Ge_0.97_Sn_0.03_ samples formed at *T*_g_ = 450 °C as a function of *T*_d_, where *T*_g_ = 450 °C. (c) *p* and (**d**) *μ*_p_ for Ge_1−*x*_Sn_*x*_ samples for *T*_d_ = 50 and 125 °C as a function of *x*, where *T*_g_ = 450 and 475 °C.

We used Hall-effect measurements to evaluate the electrical properties of the SPC-Ge_1−*x*_Sn_*x*_ layers. All samples showed p-type conduction, similar to conventional undoped poly-GeSn^45,46^. This is because the vacancy in Ge provides shallow acceptor levels that generate holes at room temperature^47^. Figure 4(a) shows that Ge_0.97_Sn_0.03_ samples have lower hole concentration *p* than Ge samples for all *T*_d_. Figure 4(b) shows that Ge_0.97_Sn_0.03_ has higher hole mobility *μ*_p_ than Ge for all *T*_d_, whereas Ge_0.97_Sn_0.03_ has smaller grains than Ge for *T*_d_ > 75 °C [Fig. 2(i)]. Figure 4(c,d) show that the electrical properties of Ge_1−*x*_Sn_*x*_ are influenced by *x*, *T*_d_, and *T*_g_. Figure 4(c) shows that Sn doping effectively lowers *p*, except for the samples with *x* = 4.5% and *T*_d_ = 125 °C. When *T*_g_ = 450 °C and *x* > 0, the samples with *T*_d_ = 50 °C exhibit lower *p* than the samples with *T*_d_ = 125 °C. In particular, the sample with *T*_d_ = 50 °C and *x* = 4.5% exhibits the lowest *p* of 1.4 × 10^17^ cm^−3^, which is the minimum *p* among poly-Ge(Sn). For *T*_d_ = 125 °C, higher *T*_g_ leads to lower *p*. This behavior is common in poly-Ge, which suggests that vacancies can be reduced by high-temperature annealing^14,30^. Figure 4(d) shows that, for all three samples, *μ*_p_ increases with increasing *x* and peaks at *x* = 3.2%. The reason why both *p* and *μ*_p_ increase and decrease at *x* = 4.5% is likely because of the decrease in crystalline quality caused by significant Sn precipitation, as suggested by Figs 2(j) and 3(b). The samples with *T*_d_ = 125 °C exhibit significantly higher *μ*_p_ than the sample with *T*_d_ = 50 °C. Furthermore, the higher *T*_g_ provides a higher hole mobility *μ*_p_. Consequently, the sample with *x* = 3.2%, *T*_d_ = 125 °C, and *T*_g_ = 475 °C exhibits the maximum *μ*_p_ of 380 cm^2^/V s.

**Figure 5:**
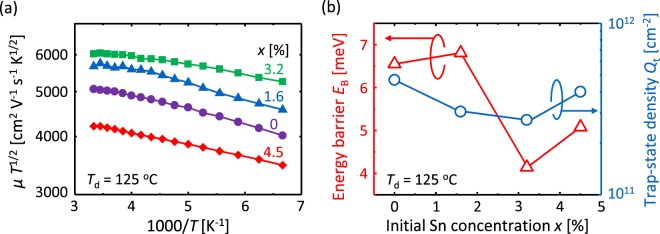
Electrical characterization of grain boundaries in SPC-Ge_1−*x*_Sn_*x*_ layers. (**a**) Arrhenius plot of *µT*^1/2^ for samples with *x* = 0, 1.6, 3.2, and 4.5%. (**b**) Energy barrier *E*_B_ and trap-state density *Q*_t_ for grain boundaries as a function of *x*. Here, *T*_d_ = 125 °C and *T*_g_ = 475 °C.

Considering that grain-boundary scattering is one factor behind decreased mobility, the hole mobility of Ge approaches the trend of grain size in Fig. 2(i). In contrast, the hole mobility of Ge_0.97_Sn_0.03_ does not follow this trend. These results suggest that *μ*_p_ of GeSn is strongly influenced by factors other than grain size. According to the carrier conduction model proposed by Seto for polycrystalline semiconductors^9^, the carrier mobility limited by grain-boundary scattering can be determined by using

3 $$\mu {T}^{1/2}=\frac{{\rm{Lq}}}{\sqrt{2{\rm{\pi }}{m}^{\ast }\,k}}\exp (-\frac{{E}_{{\rm{B}}}}{{kT}})$$

where *µ* is the carrier mobility, *E*_B_ is the energy barrier of the grain-boundary, *T* is the absolute temperature, *L* is the grain size, m^*^ is the effective mass, and *k* is the Boltzmann constant. Figure 5(a) shows that the Arrhenius plot of *µT*^1/2^ makes almost-downward-sloping straight lines for all *x*; however, the lines are slightly curved at high temperatures (1000/*T* < 4) for *x = *0‒3.2%. The trap-state density *Q*_t_ in the grain boundaries can be determined by using^8^

4 $${Q}_{t}=\frac{\sqrt{8{\rm{\varepsilon }}{N}{E}_{B}}}{q}$$

where *N* is the carrier concentration, *ε* is the dielectric permittivity, and *q* is the elementary charge. Figure 5(b) shows that *E*_B_ and *Q*_t_ determined by the slope of *µT*^1/2^ at low temperatures (1000/*T* > 4) depend on *x*. Note that the minimum values of *E*_B_ and *Q*_t_ are obtained for the highest *μ*_p_ sample with *x* = 3.2% at *T*_d_ = 125 °C.

## Discussion

The hole mobility of SPC-Ge_1−*x*_Sn_*x*_ strongly depends on both *x* and *T*_d_: a maximum mobility of 380 cm^2^/V s occurs for the sample with *x* = 3.2%, *T*_d_ = 125 °C, and *T*_g_ = 475 °C [Fig. 4(d)]. The reason is discussed as follows. According to Matthiessen’s rule and Irvin’s curve of poly-Ge with *p* on the order of 10^17^ cm^−3^ ^1^, when *μ*_p_ is less than about 250 cm^2^/V s, it is primarily limited by grain-boundary scattering as well as ordinary polycrystals. Conversely, when *μ*_p_ exceeds 250 cm^2^/V s, impurity scattering influences *μ*_p_ in addition to grain-boundary scattering. For *T*_d_ = 50 °C, *μ*_p_ increases from 80 to 250 cm^2^/V s because of Sn incorporation [Fig. 4(d)], which is attributed to the reduction of grain-boundary scattering, because of increased grain size [Fig. 2(j)] and likely because of the reduction of *E*_B_. By increasing *T*_d_ to 125 °C, *μ*_p_ increases for all *x*, reaching approximately 300 cm^2^/V s for *x* < 4.5%. We examined the effect of *T*_d_ in our previous study on SPC-Ge^11^: substrate heating at appropriate temperature during precursor deposition (*T*_d_ = 125 °C) reduces *E*_B_ and dramatically enhances *μ*_p_. This is explained from the perspective of the atomic density of the amorphous precursor. Moreover, increasing *T*_g_ to 475 °C further improves *μ*_p_ for all *x* [Fig. 4(d)]. This is likely due to the reduction of impurity scattering because of decreased *p* [Fig. 4(c)], which corresponds to the reduction of vacancy-related defects^14,30^. Even for *T*_d_ = 125 °C, *μ*_p_ peaks at *x* = 3.2% [Fig. 4(d)], although the grain size is relatively small [Fig. 2(g)]. The high *μ*_p_ is attributed to reduced impurity and grain-boundary scattering due to the decrease in *p* [Fig. 4(c)] and *E*_B_ [Fig. 5(b)], respectively. The decrease in *p* is likely caused by Sn passivating the vacancy in Ge, as mentioned in previous studies^37,46^. Meanwhile, the SPC of a-GeSn progresses while sweeping Sn, which cannot be solid-solved, to the growth front^43^. Considering that the reduction of *E*_B_ is possibly due to Sn existing at the grain-boundary, the Sn may passivate dangling bonds and thereby reduce *Q*_t_ and *E*_B_ [Fig. 5(b)]. Because excessively large *x* (>3.2%) deteriorates crystal quality [Fig. 2(j)] and *μ*_p_ [Fig. 4(d)] due to Sn precipitation, *x* should be slightly larger than the solubility limit. Therefore, the reduction of both *E*_B_ and *p*, by controlling *x* and *T*_d_, leads to the maximum mobility of 380 cm^2^/V s.

In conclusion, the precursor conditions of both the initial Sn concentration *x* and the deposition temperature *T*_d_ strongly influence the crystalline quality and electrical properties of SPC-Ge_1−*x*_Sn_*x*_. We obtain a grain size of approximately 7 μm for *x* = 1.6% and *T*_d_ = 50 °C, which is the maximum value reported to date for semiconductor films formed by SPC. Conversely, the hole mobility *μ*_p_ of GeSn reflects the energy barrier *E*_B_ and the hole concentration *p* rather than the grain size. The sample with *x* = 3.2% and *T*_d_ = 125 °C has *E*_B_ = 4.1 meV and *p* = 2.1 × 10^17^ cm^−3^, resulting in *μ*_p_ = 380 cm^2^/V s, which is the highest hole mobility among semiconductor layers formed on insulators at less than 500 °C. Since the performance of Ge-TFTs is limited by the properties of poly-Ge thin films, such high *μ*_p_ and low *p* will directly improve the field effect mobility and leakage current in the Ge-TFTs. Thus, by controlling *x* and *T*_d_ in the Ge_1−*x*_Sn_*x*_ precursor for SPC, an excellent semiconductor thin film forms at low temperature. The process developed herein is simple enough for practical fabrication of high-speed TFTs for advanced system-in-displays or three-dimensional integrated circuits.

## Methods

### Sample preparation

The Ge_1−*x*_Sn_*x*_ (0 ≤ *x* ≤ 0.12) precursors were deposited on SiO_2_ glass substrates by using the Knudsen cells of a molecular beam deposition system (base pressure of 5 × 10^−7^ Pa). The deposition rate of Ge was fixed at 1.0 nm/min, whereas that of Sn was adjusted to obtain the targeted GeSn composition. The deposition time was 100 min. The Ge and Sn source, manufactured by Furuuchi Chemical Corporation, had a purity of 99.999% and 99.9999%, respectively. The substrate temperature *T*_d_ during the deposition ranged from 50 to 200 °C. Note that *T*_d_ spontaneously rises from room temperature to 50–60 °C without heating the substrate because of the thermal energy radiated from the Knudsen cell, and the notation for this temperature is simplified as *T*_d_ = 50 °C. The samples were then loaded into a conventional tube furnace in a N_2_ atmosphere and annealed for 5 h at 450 or 475 °C to induce SPC.

### Material characterization

Rutherford backscattering spectrometry was used to determine *x* in Ge_1−*x*_Sn_*x*_ to be 0, 0.4, 0.8, 1.6, 3.2, 4.5, and 12.0%. XRR was done by using a Rigaku SmartLab, and Raman spectroscopy was done by using a Photon Design RSM-310 with a laser wavelength of 532 nm. The EBSD analyses were done by using a JEOL JSM-7001F with a TSL OIM analysis attachment. The Hall effect was measured by using the Van der Pauw method with a Bio-Rad HL5500PC. The hole mobility and hole concentration were averaged over five measurements for each sample.
